# Antioxidant Activity, Phenolic Acid, and Flavonoid Composition of an Antiseptic Ointment Based on Aloe and Green Propolis and Its Potential for Preventing Mastitis in Dairy Cows

**DOI:** 10.3390/vetsci12030248

**Published:** 2025-03-05

**Authors:** Sílvia Cristina de Aguiar, Solange Maria Cottica, Silvério Teixeira dos Santos, Juliana Maxiano da Fonseca, Luiza da Silva Leite, Mylena Leite da Silva

**Affiliations:** 1 Department of Animal Science, Universidade do Estado de Mato Grosso—UNEMAT, Pontes e Lacerda 78250000, Brazil; silverio.teixeira@unemat.br (S.T.d.S.); maxiano.fonseca@unemat.br (J.M.d.F.); mylena.leite@unemat.br (M.L.d.S.); 2 Programa de Pós-Graduação em Processos Químicos e Biotecnológicos, Universidade Tecnológica Federal do Paraná, Toledo 85902490, Brazil; smcottica@utfpr.edu.br; 3 Bioprocess Engineering and Biotechnology, Universidade Tecnológica Federal do Paraná, Toledo 85902490, Brazil; luizaleite@alunos.utfpr.edu.br

**Keywords:** antimicrobial activity, *Baccharis dracunculifolia*, mastitis prevention, milk, phenolic compounds, somatic cell count

## Abstract

Mastitis is a major challenge in dairy production, leading to decreased milk yield and quality due to increased somatic cell counts (SCCs). The overuse of antibiotics to treat this condition has raised concerns about antimicrobial resistance, prompting the search for alternative treatments. This study aimed to evaluate the antioxidant activity, phenolic acid, and flavonoid content of a natural antiseptic ointment based on green propolis and aloe vera. The phenolic and flavonoid composition was determined using ultra-high-performance liquid chromatography, revealing high concentrations of bioactive compounds, particularly in the green propolis extract. The antioxidant activity was assessed using the DPPH method, and the ointment was applied as a post-dipping treatment in dairy cows. Over a 56-day period, the SCC in cows treated with the ointment showed a significant reduction (*p* = 0.021) compared to the control group (1.94 and 2.21 log_10_ SCC/mL, respectively). These findings indicate that the combination of green propolis and aloe vera possesses promising antimicrobial and healing properties, making it an effective alternative for mastitis prevention in dairy cows.

## Simple Summary

Mastitis is a common issue in dairy cows that causes inflammation of the udder, leading to reduced milk production and quality. This condition is typically treated with antibiotics, but their overuse can lead to antibiotic resistance, which is harmful to both cows and humans. To help solve this problem, we developed a natural antiseptic ointment made from green propolis and aloe vera, which has known antimicrobial and healing properties. The results show that the cows treated with the ointment had fewer somatic cells, suggesting that the ointment effectively reduced the risk of mastitis. This natural alternative could reduce the need for antibiotics in dairy farming, helping to combat the rise of antibiotic-resistant bacteria.

## 1. Introduction

Mastitis is characterized by inflammation of the mammary gland, resulting in decreased milk production, which can be temporary or extend until the end of lactation [1]. This decline in production occurs due to the damage caused by microorganisms to mammary tissues, increasing the somatic cell count (SCC) in the milk as a result of the rise in leukocytes during infection and cell death [2]. The main etiological agents of mastitis are bacteria belonging to the genera *Staphylococcus*, *Streptococcus*, and *Escherichia coli*, with *Staphylococcus aureus* being one of the most prevalent and difficult-to-control pathogens due to its ability to form biofilms and develop resistance to antimicrobials. *Streptococcus uberis* and *Streptococcus agalactiae* are also frequently isolated from mastitis cases, while *E. coli* is an environmental pathogen responsible for severe clinical infections [1].

Depending on the severity of the infection, mastitis can occur in either a clinical or subclinical form. In clinical mastitis, physiological symptoms appear, and changes in the appearance of the milk and udder are observed. In subclinical mastitis, which is the most prevalent type, symptoms are not visible. Consequently, the disease can spread within the herd without the owner’s knowledge, thereby infecting other cows and causing significant losses. An increase in SCC and the presence of the causative agent are used as methods for detecting subclinical mastitis [3].

The current treatment of mastitis relies on antibiotics and is the primary reason for the use of antibiotics in dairy cows. However, the emergence of drug-resistant strains is threatening the viability of antibiotics for mastitis treatment. Antimicrobial resistance (AMR) occurs when pathogens can overcome the effects of antibiotics that were originally effective [4]. Numerous studies on alternative treatments are being conducted to address the concern regarding bacterial resistance due to the constant use of antibiotics in dairy herds, and this field of research continues to grow.

Propolis is one of the natural products that continues to surprise the scientific community with its benefits. Propolis is a resinous material collected by worker bees from the buds and secretions of trees from various species [5]. The chemical composition of propolis depends on several factors, such as the different types of plant sources collected by the bees, the geographical origin, and the time of year it is produced. Green propolis, a bee-derived product of significant scientific interest, is recognized for its rich phenolic composition and notable antimicrobial and anti-inflammatory properties. The primary botanical source of green propolis is *Baccharis dracunculifolia* DC, popularly known as “wild rosemary”, a plant from the Asteraceae family. Its primary bioactive compound, Artepilin C, a p-coumaric acid derivative, has demonstrated high efficacy in inhibiting mastitis-associated pathogens, including antimicrobial-resistant strains [6]. Furthermore, green propolis contains flavonoids, such as quercetin and kaempferol, which contribute to its antioxidant and anti-inflammatory activity [6,7]. An herbaceous plant that has been extensively studied for its pharmacological properties is aloe (*Aloe vera* (L.) Burm. f.). The interior of its leaves is composed of parenchymatous tissue rich in polysaccharides (mucilage), which gives it a viscous consistency. Within this mucilage are its active ingredients, which consist of organic tissues, enzymes, vitamins, mineral salts, and amino acids [8]. Aloe vera extract contains polysaccharides, aloin, and aloe–emodin, bioactive compounds with wound-healing, antimicrobial, and anti-inflammatory properties [9]. Traditionally, this medicinal plant has been used to treat skin problems (burns, wounds, and anti-inflammatory processes) [10].

In this context, this study aimed to determine the composition of phenolic acids and flavonoids in a natural antiseptic made from green propolis and aloe. The study also aimed to evaluate its effect on controlling and preventing bovine mastitis by assessing SCCs in the milk of treated cows during post-dipping.

## 2. Materials and Methods

### 2.1. Formulation of the Antiseptic Ointment Based on Green Propolis and Aloe Vera

The natural antiseptic ointment based on green propolis and aloe vera was produced at the Laboratory of Food Analysis and Animal Nutrition (LAANA—UNEMAT, Pontes e Lacerda, MT, Brazil). The raw green propolis was acquired from an apiary specialized in the production and commercialization of this type of propolis. For the extraction of the active compounds from propolis and the production of the liquid extract, the “maceration” method was used, which is the most common method for extracting propolis compounds. For maceration with a hydroalcoholic solution, 70% ethanol and 30% water were used in a ratio of 1:5 (*w*/*v*), that is, one part of raw propolis for every five parts of the hydroalcoholic solution. The extraction time was 10 days at room temperature. Subsequently, the solution was stored in an amber bottle, protected from light, and later used in the preparation of the ointment. This maceration process yielded 48% solution with the extraction of approximately 8.5% total phenolics [11].

For the extraction of aloe vera gel, leaves of the *Aloe vera* species were used. The gel was extracted using the filleting method, with a methodology adapted from Maan et al. [9].

The hydroalcoholic extract of green propolis and the aloe vera gel were mixed in a 70:30 ratio (green propolis/aloe vera). Carbopol 980 was added to this mixture to thicken the ointment. Oleic acid was added to the formulation to hydrate the skin of the cows’ teats. Subsequently, the pH of the mixture was adjusted close to the biological pH, using pH indicator strips and a neutralizing solution to achieve the ideal pH. The pH adjustment ensured the final product would not irritate the cows’ teats. The resulting mixture was then packaged in plastic tubes with funnel-shaped caps for direct application to the cows’ teats. Due to the ongoing patent application process, the quantities of the ingredients used cannot be disclosed.

### 2.2. Determination of Phenolic Acids and Flavonoids

To determine phenolic acids in the hydroalcoholic extract of propolis [12], aloe vera gel, and the antiseptic ointment, ethanolic solutions of these samples (2.5 mg/mL) were prepared. First, 250 μL of these solutions was added to test tubes, where 250 μL of Folin–Ciocalteu reagent, 500 μL of a saturated sodium carbonate solution, and 4.0 mL of distilled water were also added. The solutions were incubated in the dark for 25 min and centrifuged for 10 min at 3000 rpm, and the absorbance was measured at 725 nm against a blank in a spectrophotometer. The samples were analyzed in triplicate, and calibration curves were constructed using certified standards, such as gallic acid, to quantify the total phenolic compounds. Additionally, recovery tests were performed to assess the accuracy of the analytical methods employed, ensuring that the obtained values accurately reflected the actual concentrations of the analytes in the samples. The total phenolic content was determined through a calibration curve with gallic acid and expressed as mg of gallic acid equivalent (GAE)/g of extract.

The determination of flavonoids followed the methodology proposed by [13], with some modifications. First, 500 μL of ethanolic extract (2.5 mg/mL) was pipetted into individual test tubes. After the addition of the extract, 250 μL of 5% aluminum chloride and 4.25 mL of methanol were added to the tubes. After 30 min of resting in the dark, a reading was taken in a spectrophotometer at 425 nm, using quercetin as a standard for the calibration curve. Quality control procedures were implemented during the chemical and spectrophotometric analyses to validate the results. A blank was prepared with methanol and aluminum chloride, and a calibration curve was constructed using quercetin as a standard. All analyses were performed in triplicate to ensure precision and accuracy. From the equation of the line obtained, the flavonoid content was calculated and expressed as mg of quercetin equivalent (QE)/g of extract.

### 2.3. Quantification of Phenolic and Flavonoid Profile by UHPLC-UV-VIS

The quantification of phenolic acids and flavonoids present in the aloe vera gel and the hydroalcoholic propolis extract used in the ointment formulation was performed using ultra-high-performance liquid chromatography (UHPLC) Dionex UltiMate 3000 (Thermo Fisher Scientific Inc., Waltham, MA, USA), equipped with a UV-VIS detector (VWD-3400RS), quaternary pump (LPG-3400SD), oven (TCC-3400RS), and manual injection system, installed at the Central Analytical Laboratory of the Federal University of Technology—Paraná (UTFPR, Toledo, Paraná state, Brazil). The following standards were used: caffeic acid (>98%), syringic acid (>98%), p-coumaric acid (>98%), ferulic acid (99%), 3,5-di-caffeoylquinic acid (>98%), trans-cinnamic acid (99%), quercetin (>95%), kaempferol (>97%), and kaempferide (>99%), all obtained from Sigma-Aldrich (Sigma-Aldrich Co., St. Louis, MI, USA).

Chromatographic separation was performed using a C18 column and a ZORBAX Eclipse XDB (250 mm × 4.6 mm and 5 μm), with an oven temperature of 40 ± 1 °C and an injection volume of 20 μL. The mobile phases consisted of a mixture of (A) ultrapure water acidified with 0.5% acetic acid (pH 3.03) and (B) methanol acidified with 0.5% acetic acid at a flow rate of 1 mL/min. The elution conditions were as follows: 0–40 min, 5–65% B; 40–45 min, 65–95% B; 45–50 min, 95–95% B; and 50–55 min, 95–5% B. The analyses were performed in triplicate. The quantification of the compounds was performed by comparing their respective calibration curves. Data were acquired and processed using Chromeleon™ 7 Chromatography Data System (CDS), version 7.3.1 software (Thermo Fisher Scientific Inc., Waltham, MA, USA).

### 2.4. Determination of Antioxidant Activity

To evaluate the antioxidant activity of the hydroalcoholic propolis extract, aloe vera gel, and antiseptic ointment, the DPPH (2,2-diphenyl-1-picrylhydrazyl) method was used, as described in the literature [14], with some modifications. Initially, 50 µL of ethanolic solutions of the extracts and ointment (2.0 mg/mL) were added to 3.0 mL of ethanolic DPPH solution and kept in the dark for 30 min at room temperature. Subsequently, absorbance was measured with a spectrophotometer at 517 nm, using ethanol as a blank and Trolox for constructing the calibration curve. This assay was performed in triplicate. The results are expressed in µmol of Trolox equivalent (TE) per gram of extract. The results obtained from the DPPH assay provide information on the relative antioxidant activity of different samples and help compare the antioxidant potential of different compounds or extracts.

### 2.5. Application of Antiseptic Ointment for Post-Dipping in Dairy Cows and Evaluation of Somatic Cell Count (SCC) in Milk

The experiment was conducted on a dairy farm located in the Vale de São Domingos region, Mato Grosso state, Brazil, from January to March 2023 (rainy season). The animal trial was conducted under typical dairy farm conditions to ensure the practical applicability of the antiseptic ointment. The experimental animals were 12 crossbred multiparous dairy cows in second to third lactation, kept in an extensive grazing system, and receiving commercial concentrate at the feed trough after each milking. All sanitary management practices adhered to State regulations, with mandatory vaccinations up to date, and the routine handling procedures were maintained to avoid any impact on animal welfare or milk production. Although the sample size was limited, the study design aimed to reflect real-world dairy management, with two daily milkings, strict hygiene protocols, and consistent post-dipping practices. This approach strengthens the reliability of the results, providing insights into the ointment’s potential for mastitis prevention in a commercial dairy environment. The cows were randomly assigned into two groups: cows that underwent post-dipping with the antiseptic ointment (post-dipping) and a group of cows that did not undergo post-dipping (control). It is important to note that before the start of the experiment, pre-dipping and post-dipping procedures were not performed on the cows, making this their first exposure to such management practices in the milking parlor. Two daily milkings were performed: one at 5:00 a.m. and another at 3:00 p.m. Post-dipping was carried out as follows: after milking, the milker’s hands were sanitized with 70% alcohol before and after applying the ointment to the teats to prevent the transmission of contagious mastitis. It is worth noting that the ointment was only applied after the alcohol on the milker’s hands had completely dried. Paper towels were used to clean the teats, followed by applying the antiseptic ointment to all four teats and massaging them to ensure the product was evenly distributed. Post-dipping with the antiseptic ointment was performed daily after each milking (morning and afternoon). Milk samples were collected from all cows once every 15 days. Sampling was performed on the day the experiment began, i.e., before the application of the ointment, totaling 5 samplings (1 before and 4 after the ointment application began). The experimental period lasted 56 days. Milk samples were collected from all four teats, totaling approximately 200 mL per cow. On the same day as collection, the milk samples were analyzed for SCCs, in duplicates, using a Somaticell^®^ SCC kit (Somaticell Diagnósticos, São Paulo, Brazil).

### 2.6. Statistical Analysis

The experimental design was a completely randomized design, with two treatments (control and post-dipping) and six replications per treatment, totaling 12 crossbred multiparous dairy cows. The experiment lasted 56 days, during which milk samples were collected every 15 days, resulting in five sampling points (one before and four after the application of the antiseptic ointment). The statistical analysis of the variables studied was performed using analysis of variance (ANOVA), employing SISVAR statistical software, version 5.6 [15]. Tukey’s test was applied with a 5% significance level to determine differences between treatment means, and trends were considered when the significance value was up to 10%. For the analysis of SCCs in milk, normality and the homogeneity of variances were verified to meet the assumptions of ANOVA. Since both assumptions were violated, the SCC data were log-transformed to ensure a more robust and reliable statistical analysis. This approach allowed for the accurate interpretation of the effects of the antiseptic ointment on the prevention of mastitis in dairy cows.

## 3. Results

### 3.1. Quantification of Total Phenolic Acids Present in Aloe Vera Gel, Hydroalcoholic Extract of Propolis, and Antiseptic Ointment

The total phenolic acid content was determined in the antiseptic ointment, and two active ingredients were used in its formulation (aloe vera gel and green propolis extract, Table 1). It was observed that the hydroalcoholic extract of green propolis has a higher concentration of phenolic acids, with a concentration thirty-nine times greater than the aloe vera gel. This confirms the numerous biological properties of green propolis and the ongoing interest of researchers in studying this type of propolis. Aloe vera is also a source of phenolic acids; however, it is noted that such compounds are present in greater quantities in green propolis. In the ointment developed in this study, due to the dilution of ingredients in the formulation, the total phenolic acid concentration is significantly reduced but still sufficient to act as a natural antiseptic.

### 3.2. Concentration of Flavonoids Present in Aloe Vera Gel, Hydroalcoholic Extract of Propolis, and Antiseptic Ointment

The concentration of flavonoids present in the active ingredients used in the formulation and the antiseptic ointment was also determined (Table 2). It was found that total flavonoids could not be detected in the aloe vera gel. This was likely due to the low amount of flavonoids present in the analyzed gel or because the flavonoids in this species of aloe vera were not quantifiable by the methodology used in this study. It is important to highlight that water is added to obtain the gel through filleting, meaning the extracted gel was previously diluted with water for use. This may have contributed to the non-detection of flavonoids in the analyzed samples.

Regarding the hydroalcoholic extract of propolis, flavonoids were quantified, though in much lower amounts compared to total phenolics. Phenolic compounds can be divided into flavonoids (anthocyanins, flavonols, and isoflavones) and non-flavonoids (phenolic acids). Phenolic compounds found in plants come in various structures, such as phenolic acids, coumarin derivatives, tannins, and flavonoids [16]. Therefore, this lower concentration of flavonoids was expected, as they are a subset of total phenolics. The flavonoids in the antiseptic ointment are likely derived mostly from the hydroalcoholic extract of propolis, though in reduced amounts due to the dilutions necessary for obtaining the ointment.

### 3.3. Quantification of Phenolic Acid and Flavonoids Present in Aloe Vera Gel and Hydroalcoholic Propolis Extract

The analysis of phenolic acid and flavonoid compounds in the aloe vera gel and green propolis extract demonstrates notable differences in their chemical profiles (Table 3). Caffeic acid was present in both samples, with a significantly higher concentration in the propolis extract (328.967 ± 0.577 µg/mL) compared to the aloe vera gel (0.283 ± 0.003 µg/mL). Similarly, p-coumaric acid was more abundant in the propolis extract (1764.39 ± 1.166 µg/mL) than in the aloe vera gel (0.626 ± 0.002 µg/mL). *Trans*-cinnamic acid followed this trend, with a concentration of 368.709 ± 0.761 µg/mL in the propolis extract and 0.695 ± 0.003 µg/mL in the aloe vera gel.

In contrast, certain compounds were exclusively found in one of the samples. Syringic acid (0.886 ± 0.000 µg/mL) and 3,5-di-caffeoylquinic acid (0.999 ± 0.0131 µg/mL) were detected only in the aloe vera gel, while ferulic acid (85.134 ± 0.000 µg/mL), kaempferol (90.307 ± 0.036 µg/mL), and kaempferide (552.025 ± 0.038 µg/mL) were only present in the propolis extract. Additionally, quercetin was identified in both samples, with concentrations of 6.697 ± 0.019 µg/mL in the aloe vera gel and 12.902 ± 0.000 µg/mL in the propolis extract. These findings highlight the rich phenolic content of the green propolis extract, particularly in compounds such as p-coumaric acid and kaempferide, which were absent or present at much lower levels in the aloe vera gel.

### 3.4. Antioxidant Activity of Aloe Vera Gel, Hydroalcoholic Propolis Extract, and Antiseptic Ointment

According to the results obtained, both the aloe vera gel and the hydroalcoholic propolis extract exhibit significant antioxidant capacity, with particular emphasis on the propolis extract (Table 4). This capacity is largely attributed to the phenolic compounds present in the analyzed samples. In general, phenolic compounds are multifunctional as antioxidants, as they act in several ways: neutralizing free radicals by donating a hydrogen atom from a hydroxyl (OH) group in their aromatic structure, which has the ability to support an unpaired electron through its delocalization across the entire electron system of the molecule; chelating transition metals, such as Fe^2+^ and Cu^+^; halting the propagation of free radicals in lipid oxidation; altering the redox potential of the medium; and repairing damage to molecules attacked by free radicals [17,18]. Also, this higher antioxidant capacity observed in the green propolis extract is likely due to the high concentration of phenolic compounds present in the extract, as shown in Table 3. The antiseptic ointment showed the lowest antioxidant activity (6.96 µmol TE/g); however, as previously reported, this occurred due to the dilution of the active ingredients (aloe vera gel and green propolis extract) for the ointment’s production. Even so, it can be stated that this antioxidant activity is due to the presence of propolis and aloe vera in the ointment.

### 3.5. Somatic Cell Count (SCC) in the Milk of Cows Treated with the Antiseptic Ointment During Post-Dipping

The SCC in the milk of cows not subjected to post-dipping (control) and those subjected to post-dipping with an antiseptic ointment based on green propolis and aloe vera (post-dipping) was monitored over a 56-day period (Table 5). On day 0, the SCC in the control group was 2.15 log_10_/mL, while in the post-dipping group, it was 2.34 log_10_/mL. On day 0, only milk sampling for SCC analysis was conducted, and the experiment began after this sampling (day zero).

**Figure 1:**
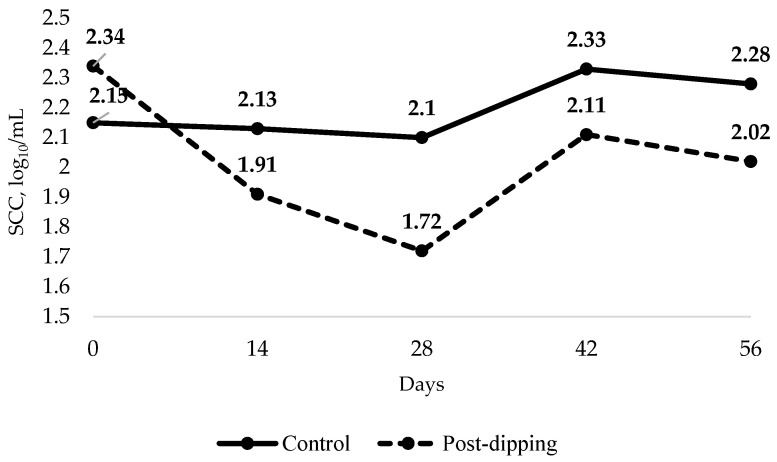
Somatic cell count (SCC) in milk from cows without post-dipping (control) and with the antiseptic ointment (post-dipping) over 56 days.

By day 14, the SCC in the control group was 2.13 log_10_/mL compared to 1.91 log_10_/mL in the post-dipping group. On day 28, the SCC in the control group was 2.10 log_10_/mL, while the post-dipping group exhibited a reduction to 1.72 log_10_/mL. By day 42, the SCC was 2.33 log_10_/mL in the control group and 2.11 log_10_/mL in the post-dipping group. Finally, on day 56, the SCC in the control group was 2.28 log_10_/mL, whereas the post-dipping group demonstrated a reduction to 2.02 log_10_/mL. These results indicate that post-dipping with the antiseptic ointment effectively reduced the somatic cell count from the first week of the experimental period (Figure 1).

The average SCC over the 56 days was significantly lower (*p* < 0.021) in the post-dipping group (1.94 log_10_/mL) compared to the control group (2.21 log_10_/mL), confirming the antiseptic efficacy of the green propolis and aloe vera-based ointment.

## 4. Discussion

The antiseptic ointment based on aloe vera gel and green propolis extract demonstrates significant biological properties, particularly related to phenolic content, flavonoids, and antioxidant activity. Each component contributes uniquely to the overall effectiveness of the formulation, supporting its antiseptic function. The phenolic content varies significantly among the aloe vera gel, green propolis extract, and the ointment (Table 1). The aloe vera gel shows a moderate phenolic concentration, while the propolis extract exhibits a strikingly higher phenolic acid content. It is known that the solvent used to extract the bioactive compounds is a determinant factor that influences the chemical composition of the obtained extracts [19]. Among the most used solvents (water, methanol, ethanol, chloroform, dichloromethane, ether, and acetone), ethanol is considered the solvent of choice for obtaining propolis extracts with a high content of bioactive compounds [20,21]. This difference suggests that green propolis is a potent source of phenolics, compounds known for their antimicrobial and antioxidant properties [22]. In comparison, the ointment shows a relatively low phenolic content, possibly due to dilution when both active ingredients are combined with other excipients. Similarly, the flavonoid content is undetectable in the aloe vera gel but significant in the green propolis extract (Table 2). Flavonoids, especially in propolis, are essential because they exhibit anti-inflammatory, antimicrobial, and antioxidant effects [23]. The antiseptic ointment retains some of these properties, though at a lower concentration.

The quantification of specific phenolic acids and flavonoids in both the aloe vera gel and propolis reveals differences in their biochemical composition (Table 3). The propolis extract contains significant amounts of caffeic acid, p-coumaric acid, and trans-cinnamic acid, which are known for their antimicrobial properties [24]. p-Coumaric acid, identified in both active ingredients (with higher concentration in the propolis extract), has proven anti-inflammatory action [25]. The authors investigated the effects of p-coumaric acid isolated from *B. dracunculifolia* on inflammation induced by lipopolysaccharide (LPS) in vivo and observed that treatment with p-coumaric not only reduced the levels of inflammatory mediators (cytokines and lipids) but also increased the production of the anti-inflammatory cytokine IL-10. In addition, the author showed that p-coumaric reduced the production of another inflammatory cytokine, IL-17, but Artepillin C, a phenolic compound marker of Brazilian green propolis, induces a stronger reduction in levels of this cytokine by inhibiting the differentiation of Th17 cells. These results confirm the potent anti-inflammatory action of green propolis, one of the goals in formulating the antiseptic ointment as a preventive agent for mastitis. In contrast, the aloe vera gel contains only trace amounts of these compounds, with syringic and 3,5-di-caffeoylquinic acid being prominent. This compositional difference underscores the potent antimicrobial activity of the green propolis extract compared to the aloe vera gel. The presence of quercetin and kaempferol, flavonoids detected in both the propolis and aloe vera samples, is notable. These compounds are known for their anti-inflammatory and antimicrobial effects [26], which contribute to the antiseptic action of the ointment.

Regarding antioxidant activity (Table 4), the aloe vera gel has substantial activity, but this is surpassed by the green propolis extract. Propolis’s higher antioxidant capacity can be attributed to its rich phenolic and flavonoid content, particularly compounds like caffeic acid and p-coumaric acid, which are known to neutralize free radicals and enhance microbial resistance [24]. However, the ointment’s antioxidant activity is significantly lower, likely due to the reduced concentration of active ingredients.

The results in Table 5 highlight the effectiveness of the antiseptic ointment, based on green propolis and aloe vera, in reducing SCCs in milk, a key indicator of udder health in dairy cows. Over a 56-day period, the SCC in cows treated with post-dipping using the antiseptic ointment decreased significantly compared to the control group, suggesting that the ointment positively impacted the reduction of mastitis, an infection characterized by elevated SCC levels. The clinical efficacy of a pharmaceutical formulation based on propolis in the prevention and treatment of bovine mastitis may lead to a reduction in the use of antibiotics and antimicrobial resistance in livestock production. After 28 days of treatment, it was found that the use of the ointment (post-dipping) showed a trend (*p* = 0.081) in decreasing SCCs (log_10_/mL). During this period, the SCC of the control group was 2.10 log_10_/mL, while in the post-dipping group, this parameter was 1.72 log_10_/mL, resulting in an 18.1% reduction in milk SCCs. Regarding the average over the experimental period (56 days), average values of 2.21 and 1.94 log_10_ SCC/mL were observed for the control and post-dipping groups, respectively, with a 12.22% reduction in SCCs in the group that received the antiseptic ointment.

Several studies report the antimicrobial activity of propolis against some bacteria that cause bovine mastitis. Loguercio et al. [27] evaluated the in vitro activity of the alcoholic extract of propolis and commonly used antimicrobials against the bacterial agents of bovine mastitis. In the study, 36 coagulase-positive *Staphylococcus* sp. isolates and 27 *Streptococcus* sp. isolates were used, and 94.4% of *Staphylococcus* sp. and 85.2% of *Streptococcus* sp. were susceptible to the propolis extract. Saeki et al. [28] isolated *Staphylococcus aureus* from the milk samples of animals with mastitis and determined its antimicrobial susceptibility profile to commercial antibiotics and the 30% propolis extract. The authors concluded that the antimicrobial effect of propolis on *Staphylococcus aureus* showed similar activity percentages (above 90%) to those of antimicrobials commonly used to treat this disease. Barbosa et al. [29] determined the in vitro inhibitory potential of propolis on the bacterium *Staphylococcus aureus* and observed that propolis has an inhibitory effect on the bacterium responsible for the studied mastitis, making it a promising alternative for in vivo studies as a replacement for conventional bactericides.

Propolis, regardless of its type, has various pharmacological properties; however, green propolis is considered the most potent due to its high concentration of Artepillin C. This compound (3,5-diprenyl-4-hydroxycinnamic acid) is a prenylated derivative of p-coumaric acid and is one of the main biologically active phenolic components of Brazilian green propolis, sourced from the plant *B. dracunculifolia*, found in the Southeast and Midwest regions of Brazil. It is known that green propolis ethanolic extract displays better antimicrobial and antioxidant activities compared to other extracts [30]. These activities may be related to the presence of Artepillin C in synergy with other constituents of the extracts [31]. Therefore, this reduction in SCCs may have occurred due to the action of compounds present in the green propolis extract, which acted as bacteriostatic agents, with Artepillin C likely enhancing this effect.

Aloe vera has also proven effective in inhibiting some microorganisms that cause mastitis. Forno-Bell et al. [32] determined whether methanolic extracts of aloe vera gel, rich in aloin A, aloin B, and aloe–emodin, have effects on the viability of bacteria responsible for mastitis in dairy cows. The results showed that treatment with aloe vera gel extract disrupted the cell membrane, causing lysis in 75% of *Staphylococcus aureus*, 88% of *Escherichia coli*, 97% of *Streptococcus uberis*, and 88% of methicillin-resistant *Staphylococcus aureus* (MRSA) cells. Another important factor to discuss is that aloe vera gel contains bioactive components (polysaccharides, aloins, and vitamins) that enhance healing both in vitro and in vivo [33,34]. Its anti-inflammatory effects reduce vasoconstriction and platelet aggregation, improving wound oxygenation, removing free radicals, and boosting collagen synthesis [35]. In the case of the antiseptic ointment, aloe vera, while contributing lesser amounts of phenolics and flavonoids than the green propolis extract, adds its well-documented skin-healing and anti-inflammatory properties to the ointment [36]. The synergy between aloe vera’s moisturizing, soothing qualities and the potent antimicrobial effects of propolis results in a comprehensive antiseptic action.

## 5. Conclusions

The antiseptic ointment based on green propolis and aloe vera shows significant potential in reducing SCC levels in milk, thereby improving udder health and reducing the risk of mastitis. The phenolic-rich composition and high antioxidant capacity of green propolis, combined with aloe vera’s soothing effects, underscore the efficacy of this ointment as an antiseptic. Though the final product contains lower concentrations of active phenolics and flavonoids than its raw components, the formulation still benefits from their antimicrobial and antioxidant properties, making it an effective solution for skin healing and infection prevention.

## 6. Patents

The antiseptic ointment based on green propolis and aloe vera is in the patent application process through the UNEMAT Innovation Agency (AGINOV).

## Figures and Tables

**Table 1:** Concentration of total phenolics in aloe vera gel, hydroalcoholic extract of propolis, and antiseptic ointment.

Sample	Phenolic Acids (mg GAE/g) ^1^	SD ^2^	CV, % ^3^
Aloe vera gel	91.18	3.75	4.11
Propolis extract	3592.95	21.56	0.60
Ointment	1.01	0.01	1.47

^1^ mg of gallic acid equivalent (GAE)/g of extract. ^2^ Standard deviation. ^3^ Coefficient of variation.

**Table 2:** Concentration of flavonoids in aloe vera gel, hydroalcoholic extract of propolis, and antiseptic ointment.

Sample	Flavonoids (mg QE/g) ^1^	SD ^2^	CV, % ^3^
Aloe vera gel	nd ^4^	-	-
Propolis extract	21.0	0.30	0.30
Ointment	0.88	0	0.51

^1^ mg of quercetin equivalent (QE)/g of extract. ^2^ Standard deviation. ^3^ Coefficient of variation. ^4^ Not detected.

**Table 3:** Quantification of phenolic compounds present in aloe vera gel and hydroalcoholic propolis extract.

Phenolic Compounds	Aloe Vera Gel	Green Propolis Extract	Retention Time (min)
	**µg/mL**		
Caffeic acid	0.283 ± 0.003	328.967 ± 0.577	12.315
Syringic acid	0.886 ± 0.000	nd	13.14
p-coumaric acid	0.626 ± 0.002	1764.39 ± 1.166	16.732
Ferulic acid	nd ^1^	85.134 ± 0.000	18.482
3,5-di-caffeoylquinic acid	0.999 ± 0.0131	nd	19.957
*Trans*-cinnamic acid	0.695 ± 0.003	368.709 ± 0.761	30.041
Quercetin	6.697 ± 0.019	12.902 ± 0.000	31.041
Kaempferol	nd	90.307 ± 0.036	35.499
Kaempferide	nd	552.025 ± 0.038	44.566

^1^ Not detected.

**Table 4:** Antioxidant activity with DPPH of aloe vera gel, hydroalcoholic extract of green propolis, and antiseptic ointment.

Sample	DPPH (µmol TE/g) ^1^	SD ^2^	CV, % ^3^
Aloe vera gel	2874.95	101.43	3.50
Propolis extract	3418.83	943.93	2.76
Ointment	6.96	0.17	2.38

^1^ Concentration in Trolox equivalents (TE)/g of extract. ^2^ Standard deviation. ^3^ Coefficient of variation.

**Table 5:** Somatic cell count (SCC) in the milk of cows without post-dipping (control) and with post-dipping using the antiseptic ointment based on green propolis and aloe vera (post-dipping) over 56 days of the experiment.

Days	Control	Post-Dipping	CV,% ^2^	*p*-Value
	SCC, log_10_/mL ^1^			
0	2.15	2.34	15.94	0.368
14	2.13	1.91	13.86	0.196
28	2.10	1.72	18.01	0.081
42	2.33	2.11	22.42	0.461
56	2.28	2.02	20.86	0.339
	Average of the experimental period (56 days)			
	2.21 ^a^	1.94 ^b^	19.94	0.021

^1^ Different lower-case letters indicate a significant difference at the 5% level (*p* < 0.05) to ANOVA. ^2^ Coefficient of variation.

## Data Availability

The data utilized and analyzed in this study can be obtained from the corresponding author upon reasonable request.
